# Teaching patient-centred communication skills during clinical procedural skill training - a preliminary pre-post study comparing international and local medical students

**DOI:** 10.1186/s12909-021-02901-7

**Published:** 2021-09-03

**Authors:** Ede Nagy, Gloria Matondo Miguel Luta, Daniel Huhn, Anna Cranz, Jobst-Hendrik Schultz, Anne Herrmann-Werner, Till Johannes Bugaj, Hans-Christoph Friederich, Christoph Nikendei

**Affiliations:** 1 Department of General Internal Medicine and Psychosomatics, University Hospital Heidelberg, University of Heidelberg, Thibautstraße 4, 69115 Heidelberg, Germany; 2 Internal Medicine VI - Psychosomatic Medicine and Psychotherapy, University of Tübingen, Tuebingen, Germany; 3 Competence Center for University Teaching in Medicine, Faculty of Medicine, University of Tuebingen, Tuebingen, Germany

**Keywords:** International medical students, Skills-lab training, Communication skills, IPPI, Binary checklist

## Abstract

**Background:**

International medical students are frequently confronted with intercultural, psychosocial, and language barriers and often receive lower marks in written, oral, and clinical-practical examinations than fellow local students. Training communication competence in procedural skills, such as blood sampling, is further challenge in this particular group of medical students. This pre-post comparative intervention study aimed to investigate the effects of training communication skills during the performance of procedural skills (taking blood samples from a silicone model) in international and local students as part of their clinical practical medical training.

**Methods:**

Study participants performed blood sampling on an arm prosthesis model (part-task trainer) before and after the communication skills training, focusing on accompanying communication with a simulation patient sitting next to the arm model. The pre- and post-evaluation video was assessed by two independent evaluators using a binary checklist, the Integrated Procedural Performance Instrument (IPPI) and global assessments of clinical professionalism in terms of procedural and communication performance. Linear models with mixed effects were used. Group differences regarding global competence levels were analysed with χ^2^-tests.

**Results:**

International medical students did not perform as well as their local counterparts in the pre- and post-examinations. Both groups improved their performance significantly, whereby the international students improved more than their local counterparts in terms of their communication performance, assessed via binary checklist. Clinical professionalism evaluated via global assessments of procedural and communication performance highlights the intervention’s impact insofar as no international student was assessed as clinically not competent after the training.

**Conclusions:**

Our results suggest that already a low-dose intervention can lead to improved communication skills in medical students performing procedural tasks and significantly increase their confidence in patient interaction.

## Background

Demographic change in many industrialized countries means that business and politics face a shortage of skilled workers. Consequently, considerable interest is being shown in attracting international educational migrants to Germany and facilitating subsequent residency [1]. Simultaneously a worldwide increase in educational migration has been observed in recent years. Increasingly, students are leaving their home countries to pursue higher education, training, and intercultural learning experiences. International students are most attracted to anglophone countries such as Australia, Canada, the United Kingdom, and the United States, as well as to destinations such as France, Germany, and the Russian Federation [2]. Every year, about 500,000 students begin their academic education at German universities. 25% of these were international students in 2019. During the winter semester 2019/2020, 12,139 students started their medical studies, whereof 2694 (22%) were international students [3].

International medical students face study-related challenges as well as psychosocial, intercultural, and language barriers [4–6]. These often include insufficient German language skills, differences in social and cultural norms as well as differences in medical practise and education. Compared to their local peers, international students report higher levels of psychological burden [7]. They are particularly anxious about falling behind in their courses, experiencing loneliness, and lacking social support, all of which often lead to increased psychological burden [5, 8]. Additionally, language barriers can cause much uncertainty regarding exam performance which can, in turn, lead to pronounced examination anxiety. Reflecting these considerations, Huhn et al. [8, 9] showed that international students actually underperform their local peers in written, oral, and practical examinations. Moreover, they were more likely to take high-stakes tests later, which is associated with extended overall study duration [10, 11]. Qualitative interviews show that international students are particularly concerned about language barriers in patient interactions [9]. Procedural skills are an essential prerequisite for future physicians’ competent patient management. Basic clinical skills, such as administering injections, central venous catheters, or stomach probes are often trained in small groups within a simulation-based setting, like skills laboratories (Skills Lab). Clinical Skills Lab training enables students to learn procedural skills in a safeguarded environment using deliberate practice and structured professional feedback [12–14].. Skills lab training has proven to be more effective than traditional bedside teaching for learning specific skills [15], shows better transfer results compared to a “see-one-do-one” approach commonly part of bedside teaching [16], and shows good results in terms of long-term retention [13]. In addition, regarding resource efficiency, peer tutors have been shown to teach skills Labs just as well as faculty members [17].

While real-life clinical procedures require both procedural and communication skills [18], skill training has traditionally focused primarily on technical expertise, ignoring other key competencies such as professionalism and communication skills. In an effort towards making simulated learning experiences more realistic, some studies have tried to bridge this gap in training accompanying communication as well as procedural skills, by creating specific clinical scenarios which combine part-task trainers with peer- [19, 20] or standardized patient- role play [21, 22]. By specifically training accompanying communication skills in a fault-forging environment prior to real life application, students are better equipped to address patient needs by means of informed clarification, accompaniment and, thus, lifting fears during clinical procedures [23]. However, to our knowledge, no studies have investigated the accompanying communication skills of international students during clinical procedural skills training to date.

The aim of the present study was to investigate the effects of an accompanying communication skills training during the performance of clinical procedural skills (blood sampling on a silicone model) in international and local students in the clinical phase of their medical studies. It was examined (1) whether international students show more deficits in communication and procedural skills compared to their fellow local students, (2) whether medical students show better objective performance in accompanying communication during clinical skills after training than before training, and (3) if international and local students differ in the improvement of their communication skills following training. We hypothesised that (1) international students would underperform local students in terms of communication skills, (2) all students would benefit from the training by showing improved objective performance in communication skills, and (3) international students were more likely to benefit from the training than their local counterparts.

## Methods

### Study design, setting & participants

In the presented pre-post comparative intervention study, participants were international and local medical students, studying at the Medical Faculty of the University of Heidelberg in the clinical phase of their studies. The study took place from December 2017 to April 2018 on the Skills Lab premises of the Medical Clinic [20]. Participants were invited via e-mail and announcements on the bulletin board in the lecture buildings of the Medical Faculty. *N* = 50 medical students registered for the study, 15 of them international and 35 local students. Before participating in the study, all interested students received a detailed information sheet introducing them to the background and objectives of the project, as well as a declaration of consent regarding their participation and the anonymised use of the collected data and video material for the study. The participants received a 20€ gift-voucher as compensation for their participation in the study. Ethical approval was granted by the Heidelberg University ethics committee (Nr. S-565/2016). Participation in the study was voluntary and all candidates were guaranteed anonymity and confidentiality. Participants’ performance in the study had no impact on assessments in their medical education. The study was conducted in accordance with the most recent version of the Declaration of Helsinki [24].

### Standardized patients

The Heidelberg University Medical Faculty has used simulation-based training with SPs for several years [25]. A total of nine SPs (six female, three male, mean age = 42.2, *SD* = 17.65; 5 years, *SD* = 4.30 of SP work experience) took part in the study. Prior to the Skills Lab trainings, the SPs received role scripts outlining their patient roles via e-mail. In addition, the SPs had a separate training session with detailed instructions on the training process and the desired reactions from them regarding the students’ actions to carefully prepare them for the actual student training [26]. For this study, their role described a patient who has to visit a general practitioner’s office every month for blood sampling because of their thyroid disease but is afraid of venous blood collection.

### Procedure

**Fig. 1:**
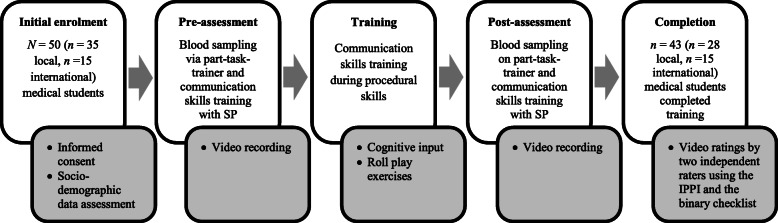
Study procedure

In total, the clinical Skills Lab training for blood sampling and accompanying communication skills took place five times, with a minimum of three and a maximum of 12 participants per session. The training itself was divided into three parts, as shown in Fig. 1. Following Kneebone et al. approach [21], the first and third part (“pre- and post-assessment”) consisted of a simulated patient encounter, in which participants’ communication skills were assessed while they took a blood sample from an artificial arm (part-task trainer) and talked to the SP sitting beside the model arm. Four separate rooms were available for pre- and post- assessment, allowing four students to perform the exercise simultaneously. The assignment of the SPs to the blood sampling stations was randomized. Both the pre- and post-assessments were digitally video recorded. Between the assessment sessions, training sessions focusing on communication skills required for clinical procedural skills were held for all participants in a separate room. This training session was designed according to the model of Maguire et al. [27] and comprised cognitive input via a PowerPoint presentation as well as an interactive display of the aforementioned role-play situation. The structure and content of the communication training are shown in Table 1.

**Table 1:** Structure and content of the communication training

**Part I: Basic Theories of Communication**	
**Definition, Meaning**	Transmission of messages; Information flow through different channels; Dialogic communication: mutual exchange in direct contact between people
**Watzlawick’s theory of communication** [28]	“You cannot NOT communicate!”
**C. Rogers’ basic attitudes in Client Centred Psychotherapy** [29]	Empathy, Appreciation, Authenticity/congruence, Transparency
**Schulz von Thun’s Four-Sides Model** [30]	(1) Case level, (2) Self-revelation, (3) Relationship level, (4) Appeal
**Conversation phases**	(1) Relationship building, (2) Problem analysis, (3) Searching for solution, (4) Agreement
**The meaning of active listening**	The emotional (affective) reaction of an interlocutor to a speaker’s message
**Conversation techniques**	Questions, Echoing, Paraphrasing, Mirroring, making pauses, Encouragement to continue talking
**Dealing with emotions**	NURSE-Model: **N**aming, **U**nderstanding, **R**especting, **S**upporting, **E**xploring
**Different communication channels**	Verbal, Non-verbal, Paraverbal
**Part II: Accompanying Communication in Clinical Practice**	
**Meaning of doctor-patient communication**	Medicine is first and foremost an interpersonal relationship
**Communication shortcomings**	Patients are interrupted in conversation; Unstructured conversations; Closed questions restrict patients; Emotional expressions are ignored; Misleading explanations from the doctor;
**Chances of good communication**	Improvement of compliance; Optimization of treatment opportunities; Extended anamnesis; New, possibly important information about patient (bio-psycho-social model)
**Interactive demonstration of the course of an ideal-typical accompanying conversation during blood sampling**	(1) Greeting/introduction, (2) Information about the procedure, (3) Clarifying open questions, (4) Asking about the patient’s well-being, (5) Clarifying experiences with previous blood samples, (6) Announcing the next steps, (7) Announcing the prick, (8) Encouraging the patient to continue talking, (9) Announcing the end, (10) Explaining the further procedure, (11) Paying attention to the patient’s well-being, (11) Allowing questions, and (12) Saying goodbye.

### Instruments

We collected the participants’ socio-demographic data and assessed their communication and clinical procedural skills during blood sampling using a binary check list [31] and the Integrated Procedural Performance Instrument [IPPI; 23].

#### Sociodemographic data

After giving informed consent, the study participants received questionnaires on socio-demographic data asking for their age, gender, current study semester, country of origin, native language, and the country in which they had obtained their highest degree of education.

#### Binary checklist

A traditional binary checklist [31] was used for evaluation of venous blood sampling performance. The checklist used in this study is regularly applied in the curriculum of the Medical Faculty of Heidelberg University Hospital and serves as a basis for OSCE (Objective Structured Clinical Examination) performance ratings [32]. The checklist-based performance rating includes 22 communication (e.g. item 6: “*Ask how the patient is feeling*”) and 12 technical-procedural items (e.g. item 17: “*Put on gloves*”). All 34 items are dichotomous items (0 = “*performed incorrectly”*, 1 = “*performed correctly”*). The individual items can be added up to a total score of the overall scale (max. 34 points) and the subscales (max. 22 resp. 12 points).

#### Integrated procedural performance instrument (IPPI) and global rating for clinical assessment of procedural performance

The IPPI was developed by Kneebone et al. [23] to evaluate clinical-practical skills in a clinical context. The instrument can be effectively used for both remote and real-time assessments in a variety of clinical scenarios. The German translation of the instrument was previously used, e.g. by Weyrich et al. [17] and Lund et al. [16]. In the present study, the assessment was based on the original 11 items on a scale of 1 to 6 (1 = “*do not agree*”, 6 = “*fully agree*”). Hereby, five items assessed communication skills (e.g. item 1: “*The student introduced him/herself to the patient and was facing the patient*”), and five items assessed clinical procedural skills (e.g. item 6: “*The student performed the puncture technically correct*”). By adding up the items, total scores were calculated for the overall scale (max. 60 points) and the two subscales (communication = 30 points; procedure = 30 points.). An additional item “*Overall ability to perform the procedure [including technical and professional skills]*” was used for clinical evaluation of the students’ procedural performance. On the basis of this item, following the suggestion of Rothman et al. [33] for the calculation of OSCE cut-off scores by using of examiners’ pass/borderline/fail judgments of candidates’ performances, this respective item served to calculate the absolute number of students rated as “competent”(5 to 6 points), “borderline” (3 to 4 points), and “not competent” (1 to 2 points).

#### Global rating for the clinical evaluation of accompanying communication skills

Since the global assessment item of the IPPI instrument refers to both the procedural and communicative competencies, we also asked the raters to get an overall impression of the students’ communication skills only as well. As part of the survey instruments, the following instruction was given to the raters: “*This item is designed to capture your overall impression of the respondent’s communication. Please indicate to what extent you agree with the statement below*. *Competent: Very good interaction with the patient; good communication skills (5 to 6 points); Borderline: Partial implementation of communicative competencies (3 to 4 points); not competent: Inappropriate interaction with patient, lack of communication skills (1 to 2 points)”.*

### Video ratings

Two interns in internal medicine residency training at the Department of General Internal Medicine and Psychosomatics at Heidelberg University Hospital independently evaluated the video recordings of medical students during patient encounters before and after training (pre- and post-assessment). The raters were trained in advance and had sessions to discuss practice video rating differences. Mean values were calculated for both assessments. Both raters were blinded with respect to design, study objective, recording date for the study, and group membership.

### Statistical analyses

Differences between international and local students regarding to gender, age, and the number of semester as well as to the global ratings of performance in the pre- and post- assessments were tested using *χ*^*2*^- and t-tests for independent samples. Interrater reliability (IRR) for the checklist and the IPP for the video ratings was calculated using the “icc” function of the “irr” package [34] for the statistical framework R [35]. Cut-offs for the agreement between raters based on ICC values are set as bad for below 0.40, as fair for values between 0.40 and 0.59, as good for values between 0.60 and 0.74, and as excellent for values between 0.75 and 1.0 [36]. To examine changes in the two study groups between T1 and T2 regarding procedural blood sampling skills, linear mixed-effects models (LMM) were applied using the “nlme” package [37] in R. LMM is more flexible than ANOVA or regression analysis with change scores as a dependent variable due to its better applicability to unbalanced design and outliers. In four different models, we specified a random intercept for individual differences in the mean of the pre- and post-assessment scores in the subscales of the binary checklist and the IPPI. Time (pre- and post-measures) and nationality (international and local students) as well as the covariates gender, age, and the number of semesters were defined as fixed effects in the models. The violation of linear model assumptions, residual normality, homoscedasticity, and the independence of explanatory variables was examined by Lilliefors (Kolmogorov-Smirnov) normality tests, QQ plots, Levene-tests, and by calculating the variance-inflation factor (VIF) as appropriate. All residuals were normally distributed, the homoscedasticity of the models was not violated, and multicollinearity was not an issue (VIF < .19). Global model fit was tested by likelihood ratio tests (“lrtest” function of the R package “lmtest” [38];). The post hoc power calculations were based on the model fits using the “Pwr”-function of the R package “nlmeU” [39].

## Results

### Sample description and response rate

Following initial enrolment, *n* = 7 students did not attend the training (dropout = 14%), so that data from *n* = 43 students could be included in the analyses. The study sample consisted of *n* = 15 international students (approximately 20% of all international medical students in the clinical study section) and *n* = 28 local students (approximately 2.5% of all local medical students in the clinical study section). Students were familiar with both communication training with peer and standardized patient role-play as well as with technical skills-training. However, this was the first session to train accompanying communication skills during clinical procedurals skills training. Sample characteristics, descriptive statistics, and comparisons of the study samples regarding to gender, age, and number of semesters are described in Table 2. There were no significant differences between the two study groups regarding gender (males = 42%, females = 48%), age (*M* = 23.43), and number of semesters (*M* = 5.8). International students came from the following countries: Syria, Kuwait, Cyprus, Cameroon (*n* = 2), Singapore, USA, Portugal, Rumania, Bulgaria, Burundi, USA, France, Italy, and Peru. Two of the international students stated that they had grown up speaking German in addition to their first language. The other students had passed a foreign student university admission language test.

**Table 2:** International and local students’ sample characteristics and tests differences

	International students (***n*** = 15)	Local Students (***n*** = 28)	Difference tests		
	*M* (*SD*) | *n (%)*	*M* (*SD*) | *n (%)*	*t* | *χ*^*2*^	*p*	*effect size*
Gender: male (=0)	5 (33.3)	12 (42.9)	0.37	.543	−0.09
Age (20–37)	23.27 (2.49)	23.43 (3.62)	0.15	.879	0.05
No. of semesters (4–13)	7 (2.48)	5.80 (1.37)	−1.76	.095	0.67

Notes: Effect sizes were determined by *phi* coefficient for gender, and *Cohen’s d* for age and semesters; for the phi coefficient .1 is considered a small effect, .3 a medium effect and .5 a large effect, and .20, .50, .80, and 1.20 for small, medium, large and very large effect for *Cohen’s d*

### Interrater reliability

Results showed a good IRR for the video ratings regarding the clinical procedural subscale of the binary checklist and a fair IRR for all other subscales. The two global items assessing clinical procedural performance and concomitant communication skills were also found to have a fair IRR (see Table 3).

**Table 3:** Interrater reliability of the binary checklist and the IPP

Subscale	***ICC***	95%-***CI*** for ***ICC***	***p***
Checklist communication	0.59	0.36 < ICC < 0.76	<.001
Checklist procedural	0.64	0.42 < ICC < 0.78	<.001
IPPI communication	0.47	0.20 < ICC < 0.65	<.001
IPPI procedural	0.46	0.19 < ICC < 0.66	<.001
IPPI overall ability	0.51	0.24 < ICC < 0.70	<.001
Global rating communication	0.44	0.16 < ICC < 0.65	.001

Notes: *ICC* = Intraclass correlation. *CI* = confidence interval. 0.40 < ICC < 0.59 = fair; 0.60 < *ICC* < 0.74 = good

### Results of binary checklists, IPPI, and clinical competence ratings

#### Mixed-effects model calculations

The results of the linear mixed-effects models are shown in Table 4. The significant likelihood ratio tests indicated a better fit of all four models with the defined covariates than the intercept only models. The explanatory variables could explain 52 and 37% of the variance of the binary checklist and the IPPI communication skill subscales. For clinical procedural subscales, variables explained 22 and 18% of the variance found in the binary checklist and the IPPI respectively.

**Table 4:** *Results of the linear mixed-effects models*

	Model: Checklist communication					Model: Checklist procedural					Model: IPPI communication					Model: IPPI procedural				
	*B*	*SE B*	*β*	*p*	*power*	*B*	*SE B*	*β*	*p*	*power*	*B*	*SE B*	*β*	*p*	*power*	*B*	*SE B*	*β*	*p*	*power*
(Intercept)	11.48	2.27	.00	<.001	1.00	9.75	1.53	.00	<.001	1.00	22.09	2.79	.00	<.001	1.00	28.34	3.06	.00	<.001	1.00
Gender	−1.08	0.64	−.18	.098	0.25	−0.52	0.43	−.16	.198	0.21	−0.73	0.78	−.10	.356	0.09	−0.25	0.86	−.04	.768	0.06
Age	0.13	0.10	.14	.194	0.34	−0.04	0.07	−.09	.489	0.07	0.01	0.12	−.01	.985	0.07	−0.20	0.13	−.20	.146	0.21
No. of semesters	0.14	0.18	.09	.420	0.12	0.10	0.12	.12	.362	0.14	0.31	0.22	.17	.163	0.28	0.32	0.24	.19	.190	0.26
Time	2.62	0.42	.52	<.001	1.00	0.62	0.26	.25	.008	0.97	2.64	0.53	.48	<.001	1.00	1.02	0.54	.25	.069	0.93
Nationality	−3.47	0.76	−.43	<.001	0.97	−1.42	0.50	−.35	.009	0.62	−3.17	0.94	−.35	.002	0.70	−2.36	1.02	−.24	.026	0.27
Time * nationality	1.47	0.70	.12	.043	0.53	0.51	0.43	.08	.303	0.21	1.52	0.90	.11	.099	0.38	1.51	0.92	.12	.108	0.36
*Model fit*																				
${R}_{\beta}^2$	0.52					0.22					0.37					0.18				
Likelihood ratio test: *χ2* (df)	68.54 (−5)			<.001		19.41 (−5)			.002		56.72 (−6)			<.001		43.09 (−5)			<.001	

*Notes: B* represents unstandardized regression weights. *SE* = standard error. *β* represents standardized regression weights calculated by the “lm.beta” function of the R package “lm.beta” [40]. ${R}_{\beta}^2$ = standardized coefficient of determination for fixed effects in the Linear Mixed-Effects Model defined by Edwards et al. [41]

#### Number of incorrect task solutions

In the binary checklist, in 47 (3.28%) out of 1432 ratings, the students were unable to solve the task in the given time, so that these respective items were rated with “0 = not completed”.

#### Checklist communication skills subscale

In the model with the sum score of the binary checklist communication skills subscale as outcome, the main effects of “time” (increase in performance in both study groups), “nationality” (local students performed better in both measurement points), and the interaction “time” x “nationality” turned out to be significant. This interaction effect indicates that, measured with the binary checklist, the international students’ communication skills increased significantly more than those of assessed local students following training. The interaction effect’s statistical power was rather low with 0.53.

#### Checklist procedural subscale

In the model with the sum score of the binary checklist procedural subscale as outcome variable, the main effects of “time” and “nationality” were significant, indicating that an increase in performance was observed in both study groups, with local students performing better at both measurement points. However, the power of the group-difference effect was relatively weak with 0.67. The interaction effect nationality x time was not significant.

#### IPPI communication skills subscale

With regard to the model with the sum score of the IPPI communication skills subscale as outcome, the main effects of “time” and “nationality” were found to be significant. This indicates an improved performance in both study groups following skills training, while local students outperformed international students at both measurement points with sufficient power. The interaction effect “time” x “nationality” revealed no significant results.

#### IPPI procedural skills subscale

In the model with the sum score of the IPPI procedural skills subscale as outcome variable, only the main effect “nationality” was significant, indicating a better performance of the local students at both measurement points. However, the power was very low with 0.27. Neither the main effect “time” nor the interaction effect time x nationality was significant.

#### IPPI overall rating for clinical assessment of procedural performance

In the pre-assessment, 27% of the international students were considered competent, 67% borderline, and 7% (*n* = 1) not competent, while 50% of the local students were considered competent and 50% borderline. In the post-assessment, 60% of the international students’ professionalism was rated as competent and 40% as borderline, while 68% of the local students’ professionalism was rated as competent and 32% as borderline and none as not competent. Therefore, the number of international students who were evaluated as competent doubled after the training, while the number of local students who were evaluated as competent only increased by 18%. Although the differences between international and local students in competency levels were smaller in the post-assessment than in the pre-assessment, these differences were not statistically significant at either pre- or post-assessment (see Table 5).

**Table 5:** Global ratings assessing students’ procedural performance (professionalism) and accompanying communication skills

Study variables (range/code)	International students (***n*** = 15)		Local students (***n*** = 28)		Difference tests					
	Pre-training	Post-training	Pre-training	Post-training	Pre-training			Post-training		
	*n (%)*	*n (%)*	*n (%)*	*n (%)*	*χ*^*2*^	*p*	*phi*	*χ*^*2*^	*p*	*phi*
IPPI - professionalism: competent (5–6)	4 (26.7)	9 (60)	14 (50.0)	19 (67.9)	2.18	.139	−0.23	.26	.606	−0.08
IPPI - professionalism: borderline (3–4)	10 (66.7)	6 (40.0)	14 (50.0)	9 (32.1)	1.10	.294	0.16	.26	.606	0.08
IPPI - professionalism: not competent (0–2)	1 (6.7)	0.0 (0.0)	0 (0.0)	0.0 (0.0)	1.91	.167	0.21	–	–	–
Global rating comm.: competent (5–6)	5(33.3)	8 (53.3)	20 (71.4)	27 (96.4)	5.82	.016	−0.37	11.98	.001	−0.53
Global rating comm.: borderline (3–4)	8 (53.3)	7 (46.7)	8 (28.6)	1 (3.6)	2.56	.110	0.24	11.98	.001	0.53
Global rating comm.: not competent (0–2)	2 (13.3)	0 (0.0)	0 (0.0)	0 (0.0)	3.91	.048	0.30	–	–	–

Notes: Effect sizes were determined by phi coefficient, where .1 is considered a small effect, .3 a medium effect and .5 a large effect

#### Global rating of competence for clinical assessment of accompanying communication skills

In the pre-assessment, 33% of the international students’ communication skills were rated as competent, 53% as borderline, and 13% (*n* = 2) as not competent, while 71% of the local students were rated as competent, 29% as borderline, and none as not competent. In the post-evaluation, 53% of the international students’ communication skills were rated as competent and 47% as borderline, while 96% of the local students’ performance was rated as competent and 4% as borderline. Both before and after the training, local students were rated as having significantly more competence compared to their international counterparts, with this difference increasing after the training. International students were significantly more likely to be classified at borderline level after training than local students, while no marked difference was found between the groups before training. Two students in the international sample were assessed as not competent in accompanying communication before training, compared to none in the local sample. This difference was statistically significant. After training, none of the students rated as not competent (see Table 5).

## Discussion

This pre-post comparative intervention study aimed to investigate the effects of training communication skills during the performance of procedural skills in international and local students as part of their clinical practical medical training. To the best of our knowledge, this is the first study to examine international students’ accompanying communication skills during clinical procedural skill training.

Results from linear models with mixed effects confirmed our first hypothesis by showing that international students underperformed in accompanying communication skills before and after training compared to local students when assessed with the communication skills subscales of both the binary checklist and the IPPI and controlling for gender, age, and number of semesters. This result is in line with previous findings, which indicate a poorer performance of international medical students in clinical-practical exams compared to local students [9]. Our second hypothesis, that both study groups would benefit from the training by significantly improved performance in accompanying communication, was also confirmed by the mixed-effect models showing excellent statistical power. Following the training, all study participants received significantly higher scores on both the communication skills subscales of IPPI and the binary checklist. A significant interaction effect between time and group was only found in the analysis of the binary checklist communication skills subscale. A similar effect was also observed in the IPPI communication skills subscale; however, the interaction term was only marginally significant here. Therefore, we could only partially confirm our third hypothesis that international students would benefit more from the training compared to their local fellow students. We used global ratings for the accompanying communication skills performance for the clinical assessment. Both before and after skills training, a larger proportion of local students were assessed as competent compared to the international subgroup. However, pointing towards the interventions positive effect on communication skills performance, no international student was classified as not competent after the training.

Regardless of whether a binary checklist [31] or the IPPI instrument [23] was used for the pre−/post- evaluation of procedural performance, the local students group outperformed the international students group. As our intervention focused on improving the students’ communication skills we did not expect any group differences regarding the improvement of procedural skills through this training. However, in terms of the binary checklist procedural skills subscale, both groups improved their post-interventional blood sampling performance compared to the pre-interventional performance scores. As this study’s intervention focused on accompanying communication and not on the training of clinical procedural skills, it is very likely that the students’ improvement in their procedural performance can be explained by repetition effects. Binary checklists [31] are designed to evaluate every step of a clinical technical procedure. The differences in procedural skill scores in this study show that the applied binary checklist is sensitive to change. With regard to the IPPI procedural skill subscale ratings, the mixed-effects model revealed a marginally significant time effect, which indicates a trend towards an improvement in procedural skills in blood sampling in both study groups. But regardless of whether the checklist or the IPPI was used, the interaction effects in the prediction of process competence remained statistically insignificant.

The global ratings of student’s professionalism [23] indicate that more students were assessed as clinically competent in procedural performance in both groups after training than before training. Furthermore, no international student was classified as not competent following the training. Consequently, supporting the clinical validity of the intervention, both groups showed improved performance in terms of correctness and professionalism in the procedural delivery of blood sampling.

Our findings demonstrate that international students’ communication skills during clinical procedural skills can be significantly improved with a brief intervention lasting little more than 1 h. Hermann-Werner et al. [13] and Lund at al [16]. could show that Skills-Lab trainings significantly impact participants’ performance directly after the training. Together, these facts have a decisive impact on international medical students’ clinical training, interprofessional teamwork in the field and patient care. Properly trained accompanying communication and improved self-confidence in communication can reduce stress for both future doctors and their patients. Accordingly, introducing regular training programs focusing on accompanying communication skills during procedural skill training might be a helpful prerequisite to help bridge the gap between Skills-Lab and real-life bed-side training. A study trying to shed light into the black box of on-ward education [42] revealed that a majority of skills performed during on-ward training goes unsupervised. This further underlines the importance of systematic, professional preparation of procedural as well as of accompanying communication skills. Our results show that one short training session is not enough to raise international students’ accompanying communication skills to the level of competence observed in their fellow local students. However, our findings suggest that more communication skills training units covering a wider range of basic procedural skills could greatly improve international students’ communication skills and, thus, their self-confidence during patient interactions.

### Limitations

The limited sample size and the unpaired study design meant that the statistical power to detect interaction effects in this study was rather low. Low statistical power increases the probability of type II error. Thus, it reduces the probability of detecting differences between groups, where differences exist. However, the clinical assessments via the global ratings of procedural performance and accompanying communication skills show the effect of this study’s intervention in that no international student was classified as not competent following training. The small sample size may also be associated with selection effects. For this reason, a re-examination of the intervention effects in a larger sample is recommended. Matching between international and local students was only done with regard to gender, age and semester of study. Future studies could also use other matching variables, such as socioeconomic status, secondary occupations, previous studies, experience in blood collection, etc. Furthermore, the time between pre- and post-assessments as well as between training and post-assessment was relatively short. Therefore, short-term memory effects may also play a role in post-test performance. Although raters were blinded in terms of group membership (local, international students), we cannot rule out the possibility that they were able to assess them as belonging to the defined group based on language skills and other characteristics. For this reason, implicit bias cannot be excluded. Longitudinal studies are needed to verify the durability of the effects achieved by the intervention. Hermann-Werner et al. [13] could show a long term benefit of a short time (90 min) Skills-Lab training regarding students’ clinical procedural performance. Consequently, we can also assume that the increased communication skills of the examined students will remain relatively stable over time. Our study did not include a non-intervention control group, making it impossible to clearly assign the effects to the intervention alone. Hence, future studies should examine the effects of accompanying communication skills training in a larger sample of medical students using a control group design. In addition, future studies should capture individual language ability to assess its impact on course material learning.

## Conclusion

In summary, our results demonstrate the effectiveness of a short intervention for accompanying communication skills in medical students. International medical students were more likely to benefit from accompanying communication skills training than their local counterparts. Consistent with previous studies, our findings highlight the direct positive impact of Skills-Lab training. Although their performance improved significantly, international medical students underperformed their local students. A short intervention can lead to improved communication skills in medical students performing procedural tasks. More training sessions covering procedural basic skills are needed to bring international students’ accompanying communication skills up to par with local students’ skills. Beyond, literature shows, that accompanying procedural skills are scarcely taught in skills-labs, why we previously proposed a model of integration role-plays into skills-lab training as also established in this training session [20]. A future integration of communication training in the curriculum is recommended in order to raise the skills of international students to the level of local students. In light of the study’s limitations, specifically the small sample size, lack of control group, and potential implicit bias, we submit that its findings are preliminary.

## Data Availability

The datasets used and analyzed during the present study are available from the corresponding author upon reasonable request.
